# Evidence for a Two-Metal-Ion Mechanism in the Cytidyltransferase KdsB, an Enzyme Involved in Lipopolysaccharide Biosynthesis

**DOI:** 10.1371/journal.pone.0023231

**Published:** 2011-08-03

**Authors:** Helgo Schmidt, Jeroen R. Mesters, Jing Wu, Ronald W. Woodard, Rolf Hilgenfeld, Uwe Mamat

**Affiliations:** 1 Institute of Biochemistry, Center for Structural and Cell Biology in Medicine, University of Lübeck, Lübeck, Germany; 2 Department of Medicinal Chemistry, College of Pharmacy, University of Michigan, Ann Arbor, Michigan, United States of America; 3 Laboratory for Structural Biology of Infection and Inflammation, DESY, Hamburg, Germany; 4 Shanghai Institute of Materia Medica, Chinese Academy of Sciences, Shanghai, China; 5 Division of Structural Biochemistry, Research Center Borstel, Leibniz-Center for Medicine and Biosciences, Borstel, Germany; Griffith University, Australia

## Abstract

Lipopolysaccharide (LPS) is located on the surface of Gram-negative bacteria and is responsible for maintaining outer membrane stability, which is a prerequisite for cell survival. Furthermore, it represents an important barrier against hostile environmental factors such as antimicrobial peptides and the complement cascade during Gram-negative infections. The sugar 3-deoxy-d-*manno*-oct-2-ulosonic acid (Kdo) is an integral part of LPS and plays a key role in LPS functionality. Prior to its incorporation into the LPS molecule, Kdo has to be activated by the CMP-Kdo synthetase (CKS). Based on the presence of a single Mg^2+^ ion in the active site, detailed models of the reaction mechanism of CKS have been developed previously. Recently, a two-metal-ion hypothesis suggested the involvement of two Mg^2+^ ions in Kdo activation. To further investigate the mechanistic aspects of Kdo activation, we kinetically characterized the CKS from the hyperthermophilic organism *Aquifex aeolicus*. In addition, we determined the crystal structure of this enzyme at a resolution of 2.10 Å and provide evidence that two Mg^2+^ ions are part of the active site of the enzyme.

## Introduction

The 8-carbon sugar 3-deoxy-d-*manno*-oct-2-ulosonic acid (Kdo) is a conserved structural element of the lipopolysaccharide (LPS) of Gram-negative bacteria. Found in the inner core regions of almost all LPS species examined to date, Kdo links the lipid A anchor to the carbohydrate domain [1]. Kdo also serves as a linker between the lipid anchor and the polysaccharide chain in group II capsular (K) antigens of pathogenic *Escherichia coli* [2]. The essential role of Kdo in maintaining outer-membrane stability as well as cell viability renders all enzymes of Kdo biosynthesis, activation and its incorporation into the maturing LPS molecule attractive targets for the discovery of novel antimicrobials [3].

**Figure 1:**
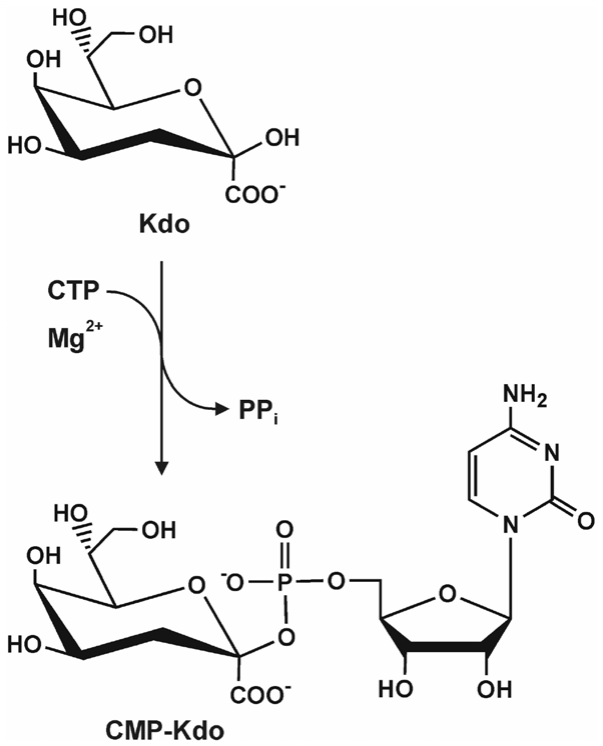
CKS reaction mechanism. Reaction scheme of Kdo activation as catalyzed by CMP-Kdo synthetase in the presence of CTP and Mg^2+^.

The Kdo pathway in Gram-negative bacteria is initiated by the enzyme d-arabinose 5-phosphate isomerase (KdsD), which catalyzes the conversion of the pentose pathway intermediate d-ribulose 5-phosphate into d-arabinose 5-phosphate (d-Ara5*P*) [4], [5]. The Kdo 8-phosphate synthetase (KdsA) subsequently condenses d-Ara5*P* with phosphoenolpyruvate to form Kdo-8-phosphate (Kdo8*P*) [6], [7], which is hydrolyzed to Kdo and inorganic phosphate by the Kdo8*P* phosphatase (KdsC) [8]. Prior to its linkage to the lipid A moiety by the Kdo transferase (WaaA), CMP-Kdo synthetase (CKS, EC 2.7.7.38) activates Kdo using CTP in a Mg^2+^-dependent reaction that results in the formation of CMP-Kdo and pyrophosphate (Fig. 1). This is the rate-limiting step in LPS biosynthesis [9], [10]. For synthesis of CMP-Kdo containing Kdo in the β-configuration, CKS utilizes the β-pyranose form of Kdo, the minor fraction of predominantly α-pyranosidic Kdo in solution [11]. The activation of Kdo to a nucleoside monophosphate diester, instead of the more common nucleoside diphosphate diester, and the extreme thermolability of the formed CMP-Kdo, with a half-life-time of 34 min at 25°C, are exceptional [12]-[14]. CMP-5-*N*-acetyl-neuraminic acid (Neu5Ac) synthetase (CNS) is the only CKS-related enzyme with a similar reaction mechanism [15]–[17]. Two CKS isozymes have been identified in *E. coli* strains that express group II K-antigens: KdsB and KpsU. Though both enzymes are homodimeric, and share 44% sequence identity, they are functionally distinct. One of the isozymes, KdsB, participates in LPS biosynthesis (LPS-specific CMP-Kdo synthetase, LCKS), while the other one, KpsU, is necessary for capsule expression (capsule-specific CMP-Kdo synthetase, KCKS) [18], [19]. Crystal structures of LCKS and KCKS from *E. coli* as well as of LCKS from *Haemophilus influenze* (HI-LCKS) have been reported [20]-[23], displaying either no or one magnesium ion in the active site. These structures show that CTP binds to a nucleotide-binding fold located in the N-terminal domain of the enzyme, whereas parts of the C-terminal domain are involved in Kdo binding and the formation of the homodimer interface. Recently, it has been suggested that the reaction mechanism of CKS enzymes resembles the two-metal-ion mechanism of DNA/RNA polymerases [20]. In the present study, we describe the enzymatic properties, the crystallization, and the X-ray crystal structure of LCKS from the hyperthermophilic organism *Aquifex aeolicus* (AA-LCKS) in complex with CTP at 2.10 Å resolution and show for the first time that two Mg^2+^ ions are present in the active site of the enzyme.

## Results and Discussion

### Biochemical characterization of AA-LCKS

Analysis of the purified AA-LCKS by SDS-PAGE revealed a monomer molecular mass of 27 kDa, while analytical gel filtration chromatography indicated an overall molecular mass of 53 kDa, approximately twice that of the monomer. We therefore conclude that AA-LCKS forms dimers, like the related CKS enzymes of *E. coli* and *H. influenzae* [20], [21], [23] and the homologous CNS of *Neiseria meningitidis* [17]. AA-LCKS was found to be active in the temperature range between 30°C and 95°C. Due to the inherent instability of CMP-Kdo, which undergoes rapid hydrolysis even at low temperatures [14], the activities measured at higher temperatures are most likely under-estimates of the true enzymatic activity. However, as expected from an enzyme of a hyperthermophilic bacterium, AA-LCKS exhibited maximum activity at 90°C. AA-LCKS was highly active within a range between pH 8.5 and 9.5, with an optimum of pH 9.0, which is in accord with the reported pH dependency of other CKS activities [9], [19], [24]. Initial rates of CMP-Kdo formation at pH 9.0 and 50°C followed Michaelis-Menten kinetics with apparent *K*_m_-values of 452±17 µM for Kdo and 160±4 µM for CTP. V_max_ was determined to be 211±11 µM min^−1^, which corresponds to a *K*_cat_ of 9.5±0.5 s^−1^. The *K*_m_ values of AA-LCKS are within the range expected on the basis of previous work on LCKS from *E. coli* (EC-LCKS) (*K*_m_-Kdo: 290 µM, *K*_m_-CTP: 200 µM) [9] and the CKS from maize (*K*_m_-Kdo: not determined, *K*_m_-CTP: 69 µM) [24], but differ significantly from recent EC-LCKS studies where *K*_m_ values of 98 µM for Kdo and 5 µM for CTP have been reported [20]. However, in the latter case, a different assay was employed to characterize EC-LCKS activity and all measurements were carried out at pH 7.5, which may account for the observed differences. Like EC-LCKS [9], AA-LCKS was able to utilize UTP for Kdo activation but not ATP or GTP. Also in the case of the CKS from maize, the successful substitution of CTP by UTP has been reported [24]. AA-LCKS Kdo turnover with UTP reached approximately 45% and 10% of the activity obtained with the regular CTP substrate when the reaction was carried out in the presence of glycine-NaOH, pH 9.0, and HEPES-NaOH, pH 7.5, respectively. The pH profile of the AA-LCKS relative activities obtained with UTP fits to the pH tendencies of UTP-dependent Kdo activation reported for other CKS enzymes [9], [24].

### Crystal structure of AA-LCKS in complex with CTP and Mg^2+^

**Figure 2:**
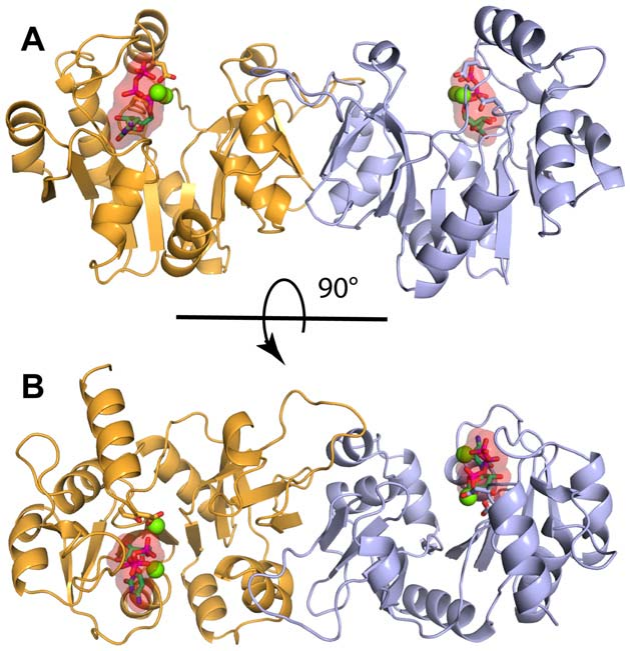
Overall structure of AA-LCKS. Cartoon diagram of the dimeric CMP-Kdo synthetase from *Aquifex aeolicus* (AA-LCKS). The CTP substrate is shown in both stick and transparent surface representation, Mg^2+^ ions are shown as green spheres. (A) Perspective highlighting the U-shaped arrangement of each protein molecule. The N-terminal domain binds the CTP substrate, whereas the C-terminal domain is responsible for Kdo binding and homodimer formation. The spatial orientation of the two domains leads to a central groove between them. (B) Top view onto the central groove. Panels (A) and (B) are related by the indicated rotation.

**Figure 3:**
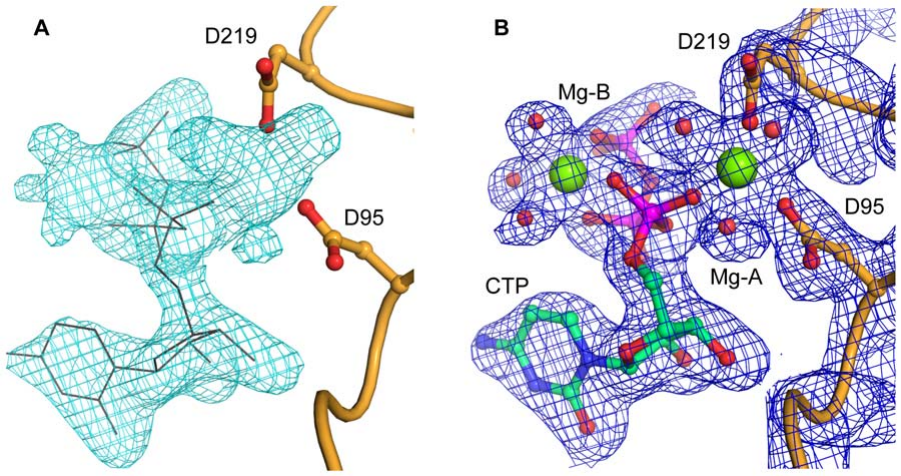
Mg^2**+**^-binding sites in AA-LCKS. (A) F_o_-F_c_ electron density of the active site of protein chain B before CTP and Mg^2+^ ions with coordinating water molecules were included in the refinement. The F_o_-F_c_ electron density is illustrated as cyan mesh and contoured at 2.5σ above the mean. For comparison, the refined CTP substrate is superposed as grey lines. (B) Final 2F_o_–F_c_ electron density after all ligands of the active site were included in the refinement. The 2F_o_–F_c_ electron density for chain B is illustrated as blue mesh and contoured at 1σ above the mean. The CTP substrate is shown in stick representation with cyan carbon and purple phosphate atoms. Water molecules as well as the Mg^2+^ ions are highlighted as spheres in red and green, respectively. In both panels, the protein main chain is shown as a ribbon. The side chains of D219 and D95 are depicted as sticks with orange carbon atoms, and nitrogen and oxygen atoms are colored in blue and red, respectively. attack.

The asymmetric unit (AU) of the AA-LCKS-CTP crystal structure contains three molecules arranged into one-and-a-half homodimers. Molecules A and B are related by a two-fold non-crystallographic symmetry axis, whereas the homodimer in the case of molecule C is formed through the crystallographic two-fold axis of spacegroup C2. Each polypeptide chain folds into a U-shaped molecule with a central, predominantly parallel, β-sheet surrounded by α-helices (Fig. 2). The overall fold of AA-LCKS is highly similar to other CKS-structures [20]–[23]. The two arms of the U-shaped molecule are formed by the N- (residues 2–91 and 220–234) and C-terminal (residues 100–210) domain, respectively, the latter being responsible for homodimer formation and Kdo binding. The three AA-LCKS molecules show no significant conformational differences. Superimposing their Cα-atoms yields low overall r.m.s.d. values in the range of 0.4 to 0.6 Å. Compared to the previously determined ternary EC-LCKS-CTP-2β-deoxy-Kdo crystal structure [20], AA-LCKS adopts a more open conformation with a larger groove between the two domains. The CTP substrate is bound to the N-terminal domain in each molecule of AA-LCKS. The protein environment interacts with the cytosine and ribose moieties of the CTP through P8, R10, L70, P71, R76, and G94 in a way already described for other CKS structures [20], [25], [26]. R10, T14, R15, and K19 of the CTP loop bind to the nucleotide's triphosphate group, which adopts a staggered conformation and harbors a Mg^2+^ ion (Mg-B) with octahedral coordination geometry (Fig. 3). The conformation of the CTP triphosphate moiety and the presence of Mg-B show some degree of variation in different CKS-CTP structures. In agreement with the situation in the AA-LCKS-CTP complex, the CTP triphosphate moiety in the EC-LCKS-CTP-2β-deoxy-Kdo crystal structure also exists in the staggered conformation and accommodates an Mg-B [20], whereas the CTP triphosphate moiety in the EC-KCKS-CTP complex shows a more extended arrangement without any indication of a bound Mg-B [26]. These observations suggest that Mg-B binding requires the staggered conformation. It has been assumed that the Mg^2+^-bound conformation represents the final CTP-binding mode prior to CMP-Kdo formation while the Mg^2+^-free one may represent a binding intermediate [20]. In the AA-LCKS-CTP crystal structure, an additional Mg^2+^ ion (Mg-A) is present between the strictly conserved side chains of D219 and D95 (Fig. 3). The octahedral coordination geometry in this case is completed by interactions with the CTP α-phosphate group and surrounding water molecules. All Mg^2+^-ligand distances in the AA-LCKS-CTP complex are in the range of 1.9 – 2.3 Å.

### Implications for the reaction mechanism

The formation of CMP-Kdo by CKS enzymes proceeds via deprotonation of the Kdo C2 hydroxyl group, followed by the nucleophilic attack of the alkoxide onto the α-phosphate of the CTP. Biochemical studies have clearly demonstrated the Mg^2+^-dependence of CKS-catalyzed reactions [9], [27], [28]. The structural work on EC-KCKS suggested the presence of a weakly bound Mg-A, which was supposed to be indirectly involved in the deprotonation of the Kdo C2 hydroxyl group via a potential hydroxide ligand, and responsible for the polarization of the attacked α-phosphorus atom of the CTP [26]. Recent work on the EC-LCKS crystal structure in complex with CTP and 2β-deoxy-Kdo revealed an Mg-B [20], but despite extensive soaking trials, no Mg-A binding to the two conserved active-site aspartic residues could be observed. Based on computational modeling and by analogy to EC-KCKS, the existence of this binding site was proposed, leading to the suggestion that EC-LCKS employs the reaction mechanism of DNA/RNA polymerases and utilizes two Mg^2+^ ions to facilitate Kdo activation [20]. The crystal structure of AA-LCKS provides the first direct structural evidence that two Mg^2+^ ions are recruited to the active site, strongly supporting the two-metal-ion scenario. Moreover, the structure demonstrates clearly that Mg-A binding does not depend on the presence of the regular Kdo substrate, as was suggested for EC-LCKS [20]. Visual inspection of the ternary EC-LCKS-CTP-2β-deoxy-Kdo crystal structure suggests that the most likely cause of the failure to produce Mg-A binding by metal ion soaking was that the CTP and the 2β-deoxy-Kdo prevented access to the conserved aspartic side-chains.

**Figure 4:**
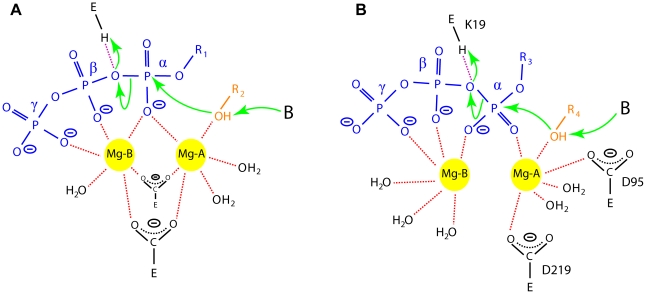
Two-metal-ion mechanism. (A) Schematic cartoon illustration of a two-metal-ion mechanism as exemplified by nucleic-acid polymerases (green arrows). The two Mg^2+^ ions (yellow) are highlighted as circles and interactions with surrounding ligands are depicted as dashed red lines. R_1_ represents the nucleoside moiety of the nucleotide triphosphate (blue), R_2_ symbolizes the growing 3′-end of a DNA or RNA chain (orange), and E corresponds to the protein environment. The interaction between the 3′-hydroxyl group of the terminal nucleotide and Mg-A lowers the pK_a_-value of the ligand and allows for its deprotonation by a general base designated B. Both Mg^2+^ ions share a common α-phosphate oxygen ligand, an interaction also maintained during the pentacovalent transition state that results after the nucleophilic attack onto the α-phosphate accomplished by the deprotonated 3′-hydroxyl group. As indicated by the purple dashed line, a basic amino-acid residue might act as general acid and protonate the additional negative charge at the β-phosphate group. (B) Schematic drawing of the proposed two-metal-ion mechanism in CMP-Kdo synthetases. R_3_ represents the cytidine moiety of the CTP and R_4_ symbolizes the C_8_H_13_O_7_ portion of the Kdo sugar. The symbol and color scheme is the same as in (A). Amino-acid residues are labeled according to AA-LCKS nomenclature. Consistent with (A), the C2 hydroxyl group of the Kdo might coordinate to Mg-A, which could facilitate its subsequent deprotonation, and K19 might play the role of a general acid. In contrast to the situation in nucleic acid polymerases and as evident from the AA-LCKS crystal structure, the two metal ions do not share a common oxygen ligand and Mg-B is not in contact with the protein environment.

**Figure 5:**
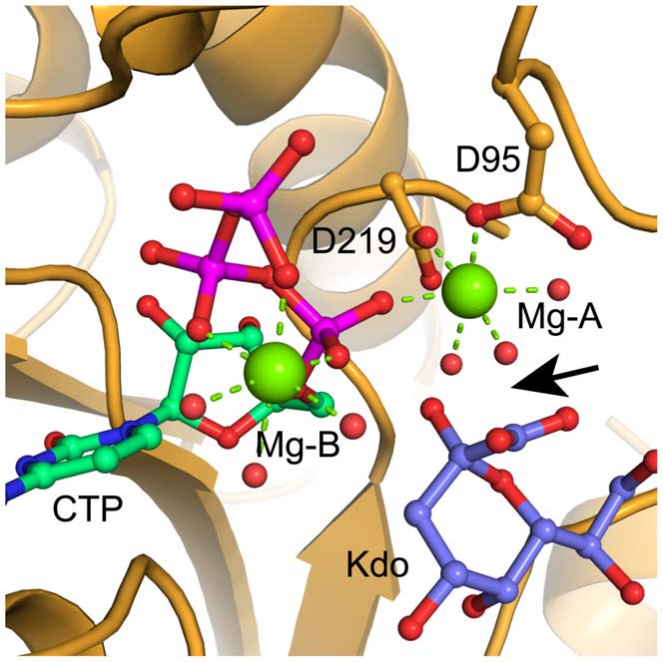
Modelled ternary AA-LCKS-CTP-Kdo complex. The CTP and Kdo substrates are shown in stick representation with cyan carbon and purple phosphate atoms (CTP) or blue carbon atoms (Kdo). Water molecules as well as the Mg^2+^ ions are highlighted as spheres in red and green, respectively. Green dashed lines indicate metal ligand interactions. The side chains of D219 and D95 are depicted as sticks with orange carbon atoms, and nitrogen and oxygen atoms are colored in blue and red, respectively. The black arrow points at steric clashes between water ligands of Mg-A and the hydroxyl and carboxyl groups of the Kdo.

In the AA-LCKS-CTP complex, a groove exists between the N- and C-terminal domain. The binding of the Kdo substrate is proposed to trigger a movement of the two domains that closes the groove and places the C2 hydroxyl group of the Kdo at an appropriate distance from the α-phosphate of the CTP for an S_n_2-type substitution reaction [20]. In a two-metal-ion mechanism [29]–[31] (Fig. 4A), Mg-A orientates/activates the nucleophile, while Mg-B, which is coordinated by oxygen atoms of the nucleotide's α-, β-, and γ-phosphate groups, is mainly responsible for the stabilization of the developing negative charge at the β-phosphate during the nucleophilic attack onto the α-phosphate. Both metal ions stabilize the transition state by coordinating to the same α-phosphate oxygen and directly interact with the protein. Most of the aforementioned two-metal-ion scenario should also apply to the situation in CKS enzymes (Fig. 4B). It has been assumed that Mg-A coordinates to the C2 hydroxyl group of the Kdo, facilitating its deprotonation, and that Mg-B helps the conserved K19 to stabilize the formation of the additional negative charge at the β-phosphate [20], [26]. A direct coordination of the C2 hydroxyl group to Mg-A might be also possible in the case of AA-LCKS as suggested by a model of the closed ternary AA-LCKS complex, which was obtained by superimposition of the N- and C-terminal domain of AA-LCKS, the ternary EC-LCKS-CTP-2β-deoxy-Kdo complex [20], and the binary HI-LCKS-Kdo complex [22]. Our model places both the C2 hydroxyl group and the carboxyl group of Kdo about 2 Å away from the Mg-A water ligands, suggesting compulsory rearrangements of the Mg-A coordination sphere during the reaction (Fig. 5). Furthermore, the modelled C2 hydroxyl group of Kdo resides in a good position for a nucleophilic S_n_2-type in-line attack on the α-phosphate of the CTP (distance ∼3 Å). In contrast to the canonical two-metal-ion-scenario (Fig. 4A), the AA-LCKS crystal structure suggests that Mg-A and Mg-B do not contact the same oxygen atom during the pentacovalent transition state of the α-phosphate. Furthermore, only Mg-A is connected to the protein via D95 and D219, while Mg-B interacts exclusively with the triphosphate moiety of the CTP and additional water molecules. The latter observation might indicate that Mg-B leaves the active site together with the pyrophosphate group, a process facilitated by the fact that this metal ion is not directly bound to the enzyme.

**Figure 6:**
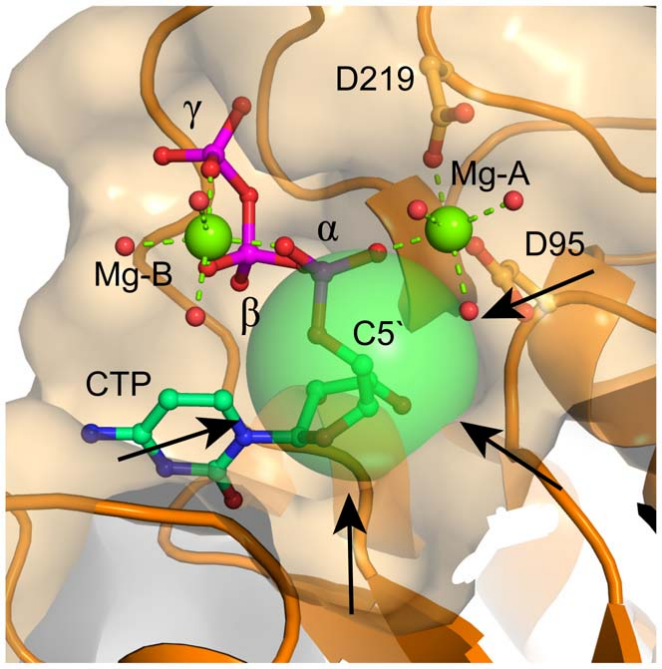
CTP-hydrolysis protection in AA-LCKS. CTP, water molecules, the Mg^2+^ ions as well as D95 and D219 are represented as described in Figure 3. Green dashed lines indicate metal-ligand interactions. The protein is shown in orange in cartoon and transparent surface representation. The transparent sphere around the C5′-methylene group features the 1.7-Å van-der-Waals radius of a carbon atom. Small nucleophiles have to access the α-phosphate from the groove between the N- and C-terminal domains, as indicated by the black arrows, and are therefore forced to pass the hydrophobic C5′-methylene group, which might lead to unfavorable interactions preventing a subsequent nucleophilic attack.

CKS enzymes not only catalyze the formation of a new covalent bond between the C2 hydroxyl group of the Kdo and the α-phosphate of the CTP, but should also prevent unproductive side reactions. Recently, it has been suggested that Mg-A binding cannot occur in the absence of the Kdo substrate, thus protecting the CTP from wasteful hydrolysis [20]. However, we observe in the AA-LCKS-CTP complex clear electron density for this nucleotide without any indication of ongoing hydrolysis, suggesting that the CTP substrate is well protected even in the presence of Mg-A and Mg-B. There are no amino-acid side-chains shielding the α-phosphate from water molecules, which could easily enter the active site and act as nucleophiles. The latter finding indicates that hydrolysis protection might be an intrinsic feature of the spatial arrangement of the CTP substrate itself. Potential nucleophiles have to access the α-phosphate exclusively from the central groove between the two domains and are therefore forced to come into close contact with the C5′-methylene group of the CTP (Fig. 6). We speculate that a small nucleophile such as a hydroxyl ion may not be able to compensate the adverse interaction with this methylene group by favorable interactions with the protein environment, which would occur in the case of the regular Kdo substrate. The orientation of the C5′-methylene group with respect to the α-phosphate might therefore be an important factor in preventing unwanted hydrolysis.

Taken together, the pH dependency of the AA-LCKS enzymatic activity, the kinetic key parameters, and the capability of AA-LCKS to utilize UTP for Kdo activation are consistent with data from other CKS enzymes, thus supporting the conclusion that the reaction mechanism of the hyperthermophilic AA-LCKS is not fundamentally different from its mesophilic counterparts. The AA-LCKS-CTP crystal structure clearly emphasizes the hypothesis that CKS enzymes follow a two-metal-ion mechanism and sheds light on mechanistic details. Unlike previously assumed, the presence of the Kdo substrate in the active site is not a prerequisite for Mg-A binding. Deviant from the classical two-metal-ion mechanism, Mg-A and Mg-B seem to be unable to coordinate to the same oxygen atom during the pentacovalent transition state of the CTP α-phosphate group and Mg-B is not in direct contact with AA-LCKS. Future research should expand on these interesting variations of the classical DNA/RNA polymerase mechanism. The findings presented here could also be of importance for the closely related CNS enzymes, which might also employ two-metal-ion catalysis for sialic acid activation [20], [32].

## Materials and Methods

### Production and purification of AA-LCKS

The recombinant production and purification of AA-LCKS was published in detail elsewhere [33]. Briefly, *E. coli* BL21-CodonPlus(DE3)-RIL cells harboring the plasmid pT7-kdsB were grown in Luria-Bertani medium supplemented with chloramphenicol (30 µg/ml) and ampicillin (100 µg/ml). Recombinant protein production was induced by adding isopropyl-β-d-thiogalactoside (IPTG) to a final concentration of 1 mM. After 3 h, the cells were harvested, resuspended in buffer A (20 mM Tris-HCl, pH 7.5, 10 mM MgCl_2_, 5 mM 2-mercaptoethanol), and disrupted by passing them through a French pressure cell. The supernatant after centrifugation was incubated at 90°C and the precipitated protein was removed by centrifugation. The protein extract was loaded onto a DEAE Sepharose FF column (GE-Healthcare) that was eluted using a linear gradient from 0 to 200 mM NaCl in the same buffer. KdsB-containing fractions were pooled and solid (NH_4_)_2_SO_4_ was added to a final concentration of 20% (w/v). The sample was applied to a Phenyl Sepharose CL-4B column (GE-Healthcare) and eluted with a reverse gradient from 20% to 0% (NH_4_)_2_SO_4_ in buffer A. The pooled KdsB-containing fractions were dialyzed against buffer A and subsequently concentrated by chromatography on a HiTrap Q Sepharose HP column (GE-Healthcare) using a linear gradient from 0 to 500 mM NaCl in buffer A. For final KdsB polishing, the pooled fractions were directly loaded onto a size-exclusion column HiLoad 26/100 Superdex 200 pg (GE-Healthcare) and eluted with buffer B (20 mM Tris-HCl, pH 7.5, 100 mM NaCl, 5 mM MgCl_2_, 2 mM 2-mercaptoethanol). The final preparation was dialyzed against buffer B, and stored at 4°C.

### Kinetic Studies

AA-LCKS activity was determined by the discontinuous assay introduced by Ray et al. [9]. A typical reaction mixture consisted of 100 mM HEPES-NaOH, pH 7.5, 10 mM MgCl_2_, 10 mM CTP, 2 mM Kdo, and 0.37 µM AA-LCKS. To obtain the temperature optimum of the enzyme, the reaction was carried out in the range between 30°C and 100°C. For the determination of the pH optimum between pH 4 and pH 10 at 80°C, HEPES-NaOH was replaced whenever appropriate by sodium acetate-acetic acid, Tris-maleate, glycine-NaOH, or borate-NaOH. V_max_ and the apparent *K*_m_ values of Kdo and CTP were determined at 50°C in the presence of 100 mM glycine-NaOH, pH 9.0. The concentration of one substrate was held constant (>5 x *K*_m_), while the other was varied over a range of 0.2 to 10 x *K*_m_. Substrates were added to the preincubated reaction mixture and after an incubation time of 1 min, the reaction was started by the addition of the enzyme and allowed to proceed for 30 s. All measurements were carried out in duplicate. *K_m_* and V_max_ were determined from a Michaelis-Menten plot using a non-linear least squares fit method. To test whether AA-LCKS is able to use other nucleotides for Kdo activation, CTP was replaced by ATP, GTP, or UTP, and the reaction was carried out at 80°C in the presence of HEPES-NaOH, pH 7.5, and glycine-NaOH, pH 9.0. Taking advantage of the appropriate temperature coefficients, all buffers have been adjusted at room temperature in such a way that the desired pH value resulted at the elevated temperatures of the kinetic experiments.

### Crystallization, data collection, and phase determination

Crystals were grown by vapor diffusion in sitting drops at 19°C. The drops contained equal amounts of KdsB (10 mg/ml in buffer B, supplemented with 1 mM CTP) and reservoir solution (0.1 M HEPES, pH 7.5, 18.8% isopropanol, 18.7% PEG 4000). The crystals belonged to space group C2 with unit-cell parameters *a* = 156.18 Å, *b* = 51.18 Å, *c* = 107.51 Å, and β = 102.68°. All crystals were mounted directly from the sitting drops into a 100-K nitrogen gas-stream (CryoStream, Oxford Instruments). Diffraction datasets were collected at EMBL beamlines X11, X13, and BW7A at Deutsches Elektronen Synchrotron (DESY) in Hamburg, Germany. The intensity data were integrated and scaled with Denzo and Scalepack [34]. Molecular replacement techniques, using the EC-KCKS-CTP crystal structure (RCSB PDB code 1H7G) [25], turned out to be unsuccessful. A comparison of 1H7G with the final AA-LCKS structure revealed substantial differences in the relative orientations of the N- and C-terminal domains, thereby preventing a molecular replacement solution for AA-LCKS. Phase information was finally obtained employing multiple isomorphous replacement with anomalous scattering after derivatization of crystals with HgCl_2_ and K_2_O_4_Os. To produce the heavy atom derivatives, crystals were soaked in reservoir solution supplemented with either 5 mM HgCl_2_ or 1 mM HgCl_2_ and 1 mM K_2_O_4_Os at 19°C for 24 – 48 h. The program Autosharp [35] in combination with Arp/wArp [36] produced a partial model that was improved by superimposing the X-ray structure with RCSB PDB code 1H7G. The improved structure was used as a search model in Molrep [37] against a SmCl_3_-derivative data set, which was chosen for further model building because of a slightly better resolution but not used for phasing. The model was completed by iterative cycles of refinement and manual model building in Refmac5 [38] and Xtalview [39]. The final protein model was refined against the native data set in Refmac5 to obtain a heavy-atom-free crystal structure. Data collection, phasing, and refinement statistics are summarized in Table 1. The atomic coordinates and structure factors were deposited with the Protein Data Bank, www.rcsb.org (RCSB PDB code 2Y6P). LSQKAB [40] of the CCP4 suite [41] was used for structural comparisons. Secondary-structure elements and hydrogen bonds were identified using DSSP [42]. The PyMOL Molecular Graphics System (http://www.pymol.org/) was used to prepare figures.

**Table 1:** Data collection, phasing, and refinement statistics.

	Native*	Hg*	Hg+Os*
**Data collection statistics**			
Wavelength (Å)	0.8416	0.803	0.803
Space group	*C*2	*C*2	*C*2
Unit cell (Å)	*a* = 156.18	*a* = 155.74	*A* = 155.44
	*b* = 51.18	*b* = 50.83	*b* = 50.93
	*c* = 107.51	*c* = 107.63	*C* = 107.38
Unit cell (°)	β = 102.68	β = 101.63	β = 101.70
Resolution (Å)	29.4 – 2.1	34.7 – 2.5	34.7 – 2.5
R_sym_^a^	4.2 (12.0)	8.8 (26.8)	8.7 (24.9)
<I>/<σI>^b^	16.8 (6.1)	14.7 (3.5)	17.0 (4.4)
Completeness (%)	91.5 (88.6)	90.8 (67.6)	92.0 (68.5)
Redundancy	3.8	5.2	6.1
n-Reflections	42413	26577	27271
**Phasing statistics**			
No. of sites	-	3	6
Phasing power^c^			
Isomorphous (acentric/centric)	-	0.878/0.825	0.877/0.701
Anomalous (acenric)	-	0.783	0.760
FOM^d^ (acentric/centric)	0.52/0.48		
**Refinement statistics**			
*R*_work_^e^ / *R*_free_^f^	21.13 / 25.79		
n-Atoms:			
Overall	5973		
Protein	5676		
Ligands	105		
Solvent	192		
r.m.s.^g^ Deviations:			
Bond lengths (Å)	0.012		
Bond angles (°)	1.375		
B-Factors (Å^2^):			
Average	40.2		
CTP	38.4		
Mg^2+^	40.0		
H_2_O	25.3		

*The values in the parentheses refer to the highest resolution shell. ^a^R_sym_ (I)  =  Σ_hkl_Σ_i_ |*I_i_(hkl)* − <*I(hkl)*>|/ Σ_hkl_Σ *I_i_(hkl)* for *n* independent reflections and *i* observations of a given reflection. <*I(hkl)*> is the average intensity of the *i* observations. ^b^<I>/<σI>, where <I> is the average intensity and <σI> is the average intensity standard deviation. ^c^Phasing power  =  (*F*_H_(calc)/*E*), where *F*_H_(calc) is the calculated heavy-atom structure factor and *E* is the estimated lack-of-closure error (isomorphous/anomalous). ^d^FOM  =  mean figure of merit. ^e^R_work_  =  Σ_hkl_ || F(obs) | − *k* | F(calc) || / Σ_hkl_ | F(obs) |. ^f^R_free_ is calculated analogous to R_work_ using 5% of the X-ray data, randomly selected for cross-validation. ^g^r.m.s.  =  Root-mean-square.
